# Microbial Communities in Soils and Endosphere of *Solanum tuberosum* L. and their Response to Long-Term Fertilization

**DOI:** 10.3390/microorganisms8091377

**Published:** 2020-09-08

**Authors:** Martina Kracmarova, Jana Karpiskova, Ondrej Uhlik, Michal Strejcek, Jirina Szakova, Jiri Balik, Katerina Demnerova, Hana Stiborova

**Affiliations:** 1 Department of Biochemistry and Microbiology, Faculty of Food and Biochemical Technology, University of Chemistry and Technology, Prague, Technicka 3, 166 28 Prague 6, Czech Republic; karpiskj@vscht.cz (J.K.); ondrej.uhlik@vscht.cz (O.U.); michal.strejcek@vscht.cz (M.S.); katerina.demnerova@vscht.cz (K.D.); 2 Department of Agro-Environmental Chemistry and Plant Nutrition, Faculty of Agrobiology, Food and Natural Resources, Czech University of Life Sciences Prague, Kamycka 129, Prague – Suchdol, 165 21, Czech Republic; szakova@af.czu.cz (J.S.); balik@af.czu.cz (J.B.)

**Keywords:** manure, sewage sludge, NPK, endophytic communities, soil microbial communities

## Abstract

An understanding of how fertilization influences endophytes is crucial for sustainable agriculture, since the manipulation of the plant microbiome could affect plant fitness and productivity. This study was focused on the response of microbial communities in the soil and tubers to the regular application of manure (MF; 330 kg N/ha), sewage sludge (SF; 330 and SF3x; 990 kg N/ha), and chemical fertilizer (NPK; 330-90-300 kg N-P-K/ha). Unfertilized soil was used as a control (CF), and the experiment was set up at two distinct sites. All fertilization treatments significantly altered the prokaryotic and fungal communities in soil, whereas the influence of fertilization on the community of endophytes differed for each site. At the site with cambisol, prokaryotic and fungal endophytes were significantly shifted by MF and SF3 treatments. At the site with chernozem, neither the prokaryotic nor fungal endophytic communities were significantly associated with fertilization treatments. Fertilization significantly increased the relative abundance of the plant-beneficial bacteria *Stenotrophomonas*, *Sphingomonas* and the arbuscular mycorrhizal fungi. In tubers, the relative abundance of *Fusarium* was lower in MF-treated soil compared to CF. Although fertilization treatments clearly influenced the soil and endophytic community structure, we did not find any indication of human pathogens being transmitted into tubers via organic fertilizers.

## 1. Introduction

The application of fertilizers to agronomical soil is a worldwide practice used for improving crop yield. Chemical fertilizers are believed to be the cause of the improvement in crop production by up to 50% during the 20th century [1]. Simultaneously, due to the constant production of organic waste material, attention has been paid to the re-use of this waste in agriculture instead of (or together with) using chemical fertilizers [2]. Therefore, interest in how the long-term application of fertilizers influence the soil microbiome has increased over the past few decades. The influence of fertilization on endophytes of cultivated plants, and especially the relation between organic fertilizers and the occurrence of pathogens has remained much less investigated.

Soil microbial diversity is an important biological factor for the assessment of soil health, soil quality and ability to suppress diseases [3,4]. Soil microorganisms are actively involved in the cycling of nutrients, and have an impact on the dynamics of nutrient turnover [5]. They participate in the decomposition of soil organic matter [6], which releases nutrients, making them available for plants. Soil microorganisms also influence the soil formation [7] and soil quality [8]. In turn, changes in environmental conditions or soil properties can directly influence the microbial community and its functioning. For instance, fertilizers (chemical or organic) introduce required nutrients into soil [9], alter soil pH [10], water holding capacity, soil texture or cation exchange capacity [11]. All these modifications of soil physicochemical properties shape the microbial community structure [12] and metabolic activity [13], which is subsequently reflected in crop yields [14].

It is not only the soil microorganisms that play a crucial role in agronomy; a similarly important role can be ascribed to the endophytes (for a review, see [15]). The microbial community of endophytes colonize inter and intracellular spaces of all plants [16]. Some endophytes live with their host-plant in a close mutualistic relationship, providing their host plant with a wide range of benefits, while the plants provide them with a protected environment and nutrients [17]. The endophytic microbes can produce substances with antimicrobial or insecticidal effects, phytohormones altering the plant growth, iron chelators, siderophores and organic acids solubilizing phosphate complexes, or they are able to fix the atmospheric nitrogen [15,16,18] and modulate the growth of roots [19]. The presence of specific beneficial endophytes can also lead to enhancement of the nutritional composition of planted crops and their vitality [20]. Thus, the alteration of endophytic communities due to the changes in soil properties (e.g., via fertilization) can have broad consequences on host plant health and growth [21,22] as well as on post-harvest storage stability [23].

The first study on the impact of agricultural practices on endophytes [24] showed a significant association between fertilization and endophytic community structure. High-nitrogen fertilization was found to increase the abundance of methanogenic archaea in plant roots, while the application of low-N fertilizer caused increases in various functional genes for nutrient metabolism in the endophyte community [21], and organic fertilizers increased the number of diazotrophic endophytes [25]. Unfortunately, there was also evidence of antibiotic-resistant bacteria transfer from manure, an example of organic fertilizer, into plant tissues [26]. This direct transfer has raised concerns about the safety of using organic fertilizers, since they are often found to harbor human pathogens [27,28].

In this study, we evaluated the effect of 21 years of regular fertilization on prokaryotic and fungal communities in bulk soil and stem tubers of *Solanum tuberosum* L. (potatoes), one of the top five crops produced worldwide [29]. The influence of manure, sewage sludge (at two different application rates) and NPK fertilizers was studied, and the effects of these treatments were compared to each other and to the unfertilized (control) soil. The experimental fields were established at two geographical locations with different soil and climate characteristics. We hypothesized that the site characteristics will be the main driver of both soil and endophytic community structures, but we expected a significant association between fertilization and the microbial community in both soil and potatoes. We suppose that fertilized soils, due to their better macronutrient properties [11], could enhance plant-beneficial genera. On the other hand, the addition of sludge and manure can bring into the soil pathogens, which can be further transfer into the plant tissues. Therefore, the benefits of fertilization will be assessed.

## 2. Materials and Methods

### 2.1. Experimental Design, Sample Collection and Processing

In 1996, experimental field-plots were established at geographically distinct sites with different characteristics (Table 1). Since that time, the field-plots have been periodically fertilized with: i) sewage sludge (SF; 330 kg N/ha), ii) sewage sludge (SF3x; 990 kg N/ha), iii) cow manure (MF; 330 kg N/ha), iv) NPK (NPK; N-P-K nutrients were 330–90–300 kg/ha). Unfertilized soil (CF) was used as a negative control. Sewage sludge was stabilized anaerobically at 55 °C, and manure was produced by proper composting (i.e., the generated heat, up to 65 °C, during the thermophilic phase is long enough to inactivate the majority of potential pathogens). Potato (*Solanum tuberosum* L.), winter wheat (*Triticum aestivum* L.) and spring barley (*Hordeum vulgare* L.) were rotated in these fields in a three-year cycle. According to typical agricultural practice, organic fertilizers (MF, SF, SF3x) were applied to the soil before potato plowing, while chemical fertilizer (NPK) was applied regularly throughout the rotation cycle. The application rate of fertilizers was calculated based on the total nitrogen input into the soil for the whole three-year rotation period (determined by the Kjeldahl method).

Sample collection was performed in September 2017, during the period of potato (*Solanum tuberosum* L. cv. Ditta; OSEVA, AGRO Brno, spol. s.r.o.) harvesting, i.e., six months after their plowing. From each fertilized variant (CF, MF, SF, SF3x, NPK), four samples of bulk soil and four samples of tubers were collected. Each soil sample consisted of six soil sub-samples, taken with a sampler probe (1.9-cm diameter) from the topsoil layer to a depth of 20 cm. These soil sub-samples were pooled together, sieved through a 2-mm mesh and stored at −20 °C until further analyses. Each potato sample consisted of three potatoes (7–10 cm average length) harvested at the same spots as the soil samples.

The tubers were washed under running tap water to remove any adhering soil. The surface of each tuber was sterilized by submerging into 70% ethanol solution for 15 s followed by flaming according to a previous study [30]. This procedure was repeated twice, and sterilized tubers were then blotted on agar plates to verify the sterilization process.

Half of each tuber was manually disintegrated and ground under liquid nitrogen in a laminar hood. All equipment used was sterile, and the whole surface sterilization procedure was performed under aseptic conditions. Disintegrated plant material was stored at −20 °C until further analyses.

### 2.2. DNA Isolation

Metagenomic DNA was extracted from 0.5 g of soil or plant material using a FastDNA SPIN kit for soil (MP Biomedicals, Solon, OH, USA) and purified with Genomic DNA Clean and Concentrator™ (ZYMO Research, Irvine, CA, USA) following the manufacturers’ protocol. DNA concentration and purity were determined spectrophotometrically using a NanoDrop ND-1000 (NanoDrop Technologies, Wilmington, DE, USA).

### 2.3. 16S rRNA Gene and ITS Region Amplicon Generation from Soil Materials

Amplicons of soil origin were prepared using two sequential polymerase chain reactions (PCRs) with specific primers. For 16S rRNA gene amplification, 515 forward (5′-GTGYCAGCMGCNGCGG-3′) and 926 reverse (5′-CCGYCAATTYMTTTRAGTTT-3′) primers were targeted to the V4–V5 region of the gene [31]. For ITS region amplification, 5.8S_Fun forward (5′-AACTTTYRRCAAYGGATCWCT-3′) and ITS4_Fun reverse (5′-AGCCTCCGCTTATTGATATGCTTAART-3′) primers were used [32]. The master mix content and temperature program was adopted from previous study [31]. Briefly, the initial reactions of 15 µL contained: 0.02 U/µL KAPA HiFi HotStart ReadyMix (Kapa Biosystems, Wilmington, MA, USA), metagenomic DNA (~10 ng), 0.3 µM of each primer (Sigma-Aldrich, St Louis, MO, USA) and water for molecular biology (Sigma-Aldrich, St Louis, MO, USA). In the second amplification run, the product of the first PCR was used as a template DNA, and the same primers modified with adaptor tags and internal barcodes for Illumina sequencing were used. The second 25 µL reactions contained 0.02 U/µL KAPA HiFi HotStart ReadyMix (Kapa Biosystems, Wilmington, MA, USA), 1 µM of each primer (Sigma-Aldrich, St Louis, MO, USA), 0.5 µL of the previous PCR product and water for molecular biology (Sigma-Aldrich, St Louis, MO, USA). The temperature regime for both reactions was as follows: 95 °C/5 min, 98 °C/20 s, 56 °C (for 16S rRNA gene) or 50 °C (for ITS region)/15 s, 72 °C/15 s, 72 °C/5 min. The first amplification was prepared with 28–30 cycles; the next amplification was performed with 8–10 cycles.

### 2.4. 16S rRNA Gene and ITS Region Amplicon Generation from Plant Materials

Amplicons of the ITS region were prepared using the same procedure as used for soil samples. However, 16S rRNA amplicons were prepared using three sequential PCRs. In the first PCR, amplicons of the 16S rRNA gene were generated using 515 forward and 1068 reverse (5′-CTGRCGRCRRCCATGCA-3′) primers together with anti-mitochondrial and anti-plastid peptide-nucleic acids (PNAs) (PNABio, Thousand Oaks, CA, USA) to inhibit the amplification of plant organelle DNA [30]. The first 15 µL reactions contained 0.3 µM of each PNA probe, 0.3 µM of each primer (Sigma-Aldrich, St Louis, MO, USA), 0.02 U/µL of KAPA HiFi HotStart ReadyMix (Kapa Biosystems, Wilmington, MA, USA), template DNA (~10 ng/µL) and water for molecular biology (Sigma-Aldrich, St Louis, MO, USA). The amplification of each sample was performed in six copies and analyzed by agarose gel (1.5%) electrophoresis. The band at 553 bp was excised and purified using a Zymoclean Gel DNA Recovery Kit (ZYMO Research, Irvine, CA, USA).

Extracted DNA was then used in the second PCR, the composition of which and temperature regime were the same as for the first PCR described for soil samples. The product from the second PCR was used as a DNA template for the third PCR, during which the amplicons with adaptor tags and internal barcodes for Illumina sequencing were generated. The third amplification was performed according to the same procedure as described for the second PCR in soil samples.

### 2.5. Amplicon Purification and Sequencing

The resultant amplicons of 16S rRNA and ITS regions of plant and soil origin were purified with SPRIselect magnetic beads (Beckman Coulter, Miami, FL, USA) and sent on ice packs to the Core Facility for Nucleic Acid Analysis at the University of Alaska Fairbanks for Illumina Miseq platform sequencing.

Along with soil and plant samples, amplicons generated from mock community DNA standards (ZymoBIOMICS Microbial Community DNA Standard, ZYMO Research, Irvine, CA, USA) were used as a positive control and to identify potential errors from amplification steps and to obtain the proper parameters for sequence data processing.

### 2.6. Data Processing

In the program R [33], raw sequences delivered from Illumina Miseq were processed using the DADA2 package [34] with a procedure adopted from DADA2 pipeline version 1.12. After the removal of the primer sequences, the 16S rRNA gene sequences were then filtered using the following parameters: truncLen = c(227,178), maxN = 0, maxEE = c(2,2), truncQ = 2. With the ITS region, the primer reads of both orientations were identified and trimmed off, and the sequences were filtered using the following parameters: truncLen = c(0,0), maxN = 0, maxEE = 2, truncQ = 2. In both the 16S rRNA gene and ITS region datasets, chimeric sequences were identified and removed according to the “consensus” method. To reduce the potential errors introduced during sequencing, sequences that differed by one base (or by up to two bases for the ITS region) were merged, while keeping the most abundant one as the valid sequence [31]. The taxonomy was assigned using the silva_nr_v132_train_set.fa.gz [35] database for 16S rRNA gene sequences and the UNITE database [36] for ITS region sequences. All obtained MiSeq reads were deposited in the NCBI Short Read Archive under BioProject accession number PRJNA645139.

Manual inspection of the ITS region amplicon sequence variants (ASVs) with no assigned taxonomy and abundance higher than 100 reads was performed using the Basic Local Alignment Search Tool (BLAST) algorithm (National Center for Biotechnology Information, NCBI). In total, 25.9% of ITS region ASVs that were clearly of plant DNA origin (Query cover 100%, Percentage identity > 96%) were discarded. ASVs generated from 16S rRNA genes that were assigned as mitochondria at the family level or chloroplast at the order level, accounting for 41.7% of ASVs, were also removed from the dataset.

### 2.7. Multivariate Statistical Analyses

Further analyses of microbial data were conducted in R using the packages *phyloseq* [37] and *vegan* [38]. Alpha diversity was assessed by calculating the Shannon diversity index [39], and the non-parametric Kruskal-Wallis test was used for testing for significant differences in microbial diversity between treatments. The analysis of changes in microbial diversity was at first conducted on the whole prokaryotic and fungal community datasets, and then separately on soil and plant samples.

Principal coordinate analysis (PCoA) based on Bray-Curtis distance was used to examine the differences in microbial communities originating from soil and potatoes, collected at two sites. Then, the datasets were split according to sample origin, into soil samples and tuber samples, and converted into relative abundance data with the Hellinger transformation [40]. The statistical significance of fertilization treatment, site characteristics and the interaction of both factors on microbial community data was determined with permutational multivariate analysis of variance (PERMANOVA) [41,42]. Pairwise PERMANOVA was conducted to test for significant differences in microbial communities in the control treatment and each fertilized variant. The false discovery rate (FDR) was used to calculate the corrected *p*-values [43], and permutations did not involve blending together samples of different sites. To compare how soil and endophytic communities were differentiated by fertilization regimes, PCoA analysis was applied on microbial data, and the PCoA ordination plot was constructed.

To identify genera with significantly different abundance in soil or tubers originating from fertilized variants compared to CF, differential abundance analysis was conducted using the *DESeq2* package [44] on non-transformed datasets. To determine statistical significance, the fold-change threshold was set to 1.2 and *p-*value with false discovery rate correction was set to 0.01.

To identify human bacterial pathogens in soil and tuber samples, the 16S rRNA gene ASVs were compared to a database of 122 bacterial pathogenic species adopted from previous study [28] using a local BLAST+ search with 99% sequence identity and expect values cutoff of 1.0e-10 [45]. The ASVs assigned to potential human pathogenic species were applied to the heat map constructed based on non-metric multidimensional scaling (NMDS) with Bray-Curtis dissimilarity for samples (*plot_heatmap* function, *phyloseq* package); a slash mark was used when one ASV was assigned to more than one bacterial species.

## 3. Results

### 3.1. Analysis of Microbial Diversity in Soil and Potatoes

**Figure 1:**
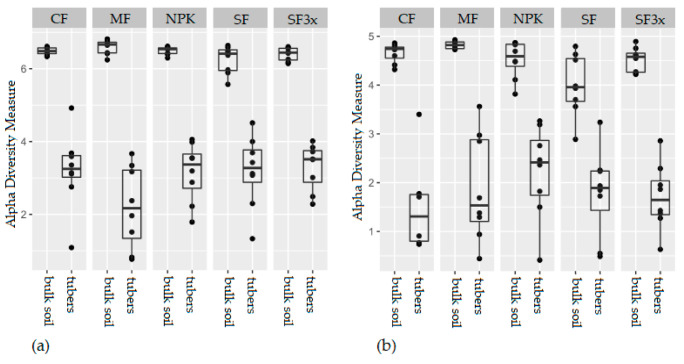
Shannon diversity index calculated from prokaryotic (**a**) and fungal (**b**) sequence data originating from bulk soil and potato samples. Samples were collected from different fertilization treatments: control (CF), manure (MF; 330 kg N/ha), NPK (NPK; 330-90-330 kg/ha), sewage sludge (SF; 330 kg N/ha), sewage sludge (SF3x; 990 kg N/ha).

In total, 10,801 unique 16S rRNA gene ASVs and 4100 unique ITS ASVs were obtained from all soil samples, and 1803 unique 16S rRNA gene ASVs and 550 unique ITS ASVs were obtained from tuber samples. Correspondingly, the Shannon diversity index (Figure 1) significantly differed between soil and tuber samples for both prokaryotic and fungal communities (*P* ≤ 0.001, Kruskal-Wallis), being higher in soil samples. There was no evidence of a significant influence of the fertilizer on the diversity of soil prokaryotes (*P* > 0.05, Kruskal-Wallis), in fact, the fertilization treatment only significantly influenced soil fungal diversity (*P* ≤ 0.05, Kruskal-Wallis). Specifically, fungal diversity with the MF treatment was significantly higher than with the SF treatment (*P* ≤ 0.05, Pairwise Wilcoxon), but the diversity did not differ significantly from the CF in any of the fertilized soils. The diversity of endophytes, both fungal and prokaryotic, was not found to be significantly associated with the fertilization treatment (*P* ≤ 0.05, Kruskal-Wallis).

### 3.2. Microbial Community Structure in Soil and Tubers

**Figure 2:**
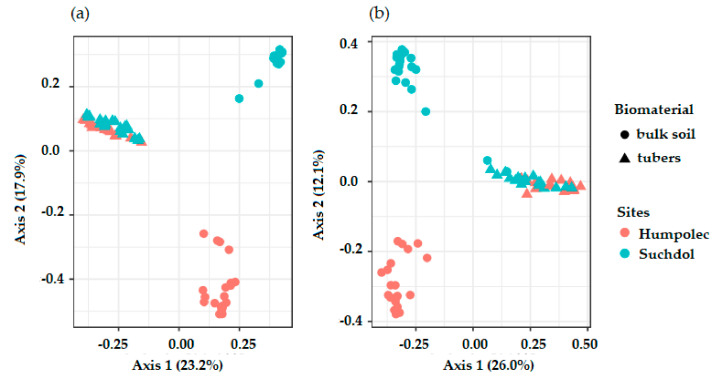
Principal coordinate analysis (PCoA) ordinations of soil and tuber samples based on prokaryotic (**a**) and fungal (**b**) amplicon sequence variants (ASV) sequence data.

Whereas microbial community structure, both prokaryotic and fungal, in the two different soils formed separated clusters in the ordination space (PCoA, Figure 2), the endophytic microbial communities grouped together (PCoA, Figure 2). Soil and endophytic communities were separated on the first PCoA axis, accounting for 23.2% and 26.0% for prokaryotes and fungi, respectively.

The community structure of both soil prokaryotes and fungi was found to be significantly associated with the site, fertilization regime and with the interaction of these factors. Among these factors (fertilization, site characteristics and their interaction), the site characteristics explained the most variability in soil prokaryotic (57%) and fungal (39%) communities (PERMANOVA, Table 2). The structure of endophytic prokaryotic communities was also significantly associated with fertilization, site characteristics and also the interaction of these factors (PERMANOVA, Table 2), while the association between the structure of endophytic fungal communities and fertilization was marginal. The interaction of fertilization and site characteristics was responsible for most of the variability in prokaryotic and fungal communities (PERMANOVA R^2^, Table 2).

### 3.3. Specific Effect of Fertilization on Microbial Community Structure

**Figure 3:**
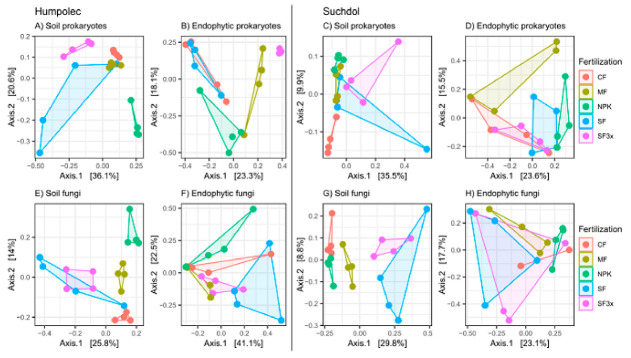
PCoA ordination demonstrating differences in prokaryotic and fungal communities in soil and tubers collected from different fertilization regimes: control (CF), manure (MF; 330 kg N/ha), NPK (NPK; 330-90-330 kg/ha), sewage sludge (SF; 330 kg N/ha), sewage sludge (SF3x; 990 kg N/ha). Subfigures represent: (**A**) soil prokaryotic communities in Humpolec, (**B**) endophytic prokaryotic communities in Humpolec, (**C**) soil prokaryotic communities in Suchdol, (**D**) endophytic prokaryotic communities in Suchdol, (**E**) soil fungal communities in Humpolec, (**F**) endophytic fungal communities in Humpolec, (**G**) soil fungal communities in Suchdol, and (**H**) endophytic fungal communities in Suchdol.

To elucidate how the soil and endophytic communities differed in response to the fertilization treatments, pairwise PERMANOVA and PCoA based on Bray-Curtis distance were conducted separately for both site characteristics. Soil prokaryotic and fungal communities at both sites significantly differed between the fertilization treatments (*P_adj_* ≤ 0.05, pairwise PERMANOVA), except for communities of SF and SF3x-treated soils. Additionally, soil prokaryotic communities in SF- and MF-treated soils in Suchdol were not found to be significantly different either. Furthermore, the microbial community structure in the sewage sludge-treated soils (SF and SF3x) were the most dissimilar from other treatments, which was shown by their separation along the first PCoA axis (Figure 3A,C,E,G).

The response of endophytic communities to fertilization treatments varied according to the site characteristics. In Humpolec, the communities of prokaryotes significantly differed in all fertilization treatments compared to CF (*P_adj_* ≤ 0.05, pairwise PERMANOVA), with an exception for the communities of CF- vs. SF-treated soils, which is also shown in the PCoA ordination (Figure 3B). Fungal communities in Humpolec were significantly shifted in SF, SF3x and MF treatments compared to the CF (*P_adj_* ≤ 0.05, pairwise PERMANOVA). Furthermore, the fungal communities of SF-treated soil significantly differed from those in MF and NPK soils, and SF3x communities differed from those in NPK soil (*P_adj_* ≤ 0.05, pairwise PERMANOVA). These results correlate with the PCoA ordinations in Figure 3F. Neither prokaryotic nor fungal endophytic communities in Suchdol were associated with fertilization treatments (*P_adj_* > 0.05, pairwise PERMANOVA). Only prokaryotes in NPK-treated tubers differed from the CF at the marginal level of significance; similarly, the prokaryotes in SF3x differed from those in MF and NPK (*P_adj_* = 0.072, pairwise PERMANOVA).

### 3.4. Genera Significantly Enriched in Fertilized Soil or Tubers

Differential analysis was conducted to identify the prokaryotes and fungi, the relative abundance of which was significantly different between CF and fertilized soils (Figures S1 and S2) and the respective tubers (Figure S3), assuming a significance threshold of *P_adj_* ≤ 0.01. These genera were grouped according to the fertilization treatments in which their abundance was higher, and the results are summarized in Table 3. In total, the relative abundance of 20 prokaryotic and seven fungal genera was increased in fertilized soils, with most of the genera being enriched by more than one type of fertilization treatment. With the exception of *Conocybe*, all of the identified prokaryotic and fungal genera were enriched in sewage sludge-treated soil (SF and/or SF3x).

Three prokaryotic genera were significantly enriched in tubers grown in fertilized soil (Table 3C), while no fungal genera with significantly higher abundance were found in tubers originating from any of the fertilization variants when compared to CF. Finally, a significantly higher abundance of *Fusarium* was detected in CF versus MF treatment (Figure S3).

### 3.5. Transmission of Potential Human Pathogens

**Figure 4:**
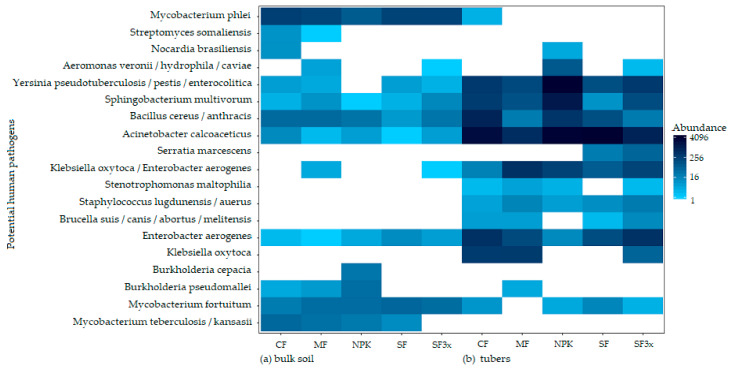
Heatmap representing abundance of 20 bacterial ASVs that were assigned to one (or more) potential human pathogens. Samples taken from soil (**A**) and tubers (**B**) originating from different fertilization treatments: control (CF), manure (MF; 330 kg N/ha), NPK (NPK; 330-90-330 kg/ha), sewage sludge (SF; 330 kg N/ha), sewage sludge (SF3x; 990 kg N/ha). A white field indicates the absence of the corresponding ASV in the sample.

In total, 22 16S rRNA gene ASVs were assigned to one (or more) potential human pathogens. Their different abundance across the samples is visualized in Figure 4. Seven of these ASVs occurred in almost all of the soil or plant samples across all treatments. These ubiquitous ASVs were assigned to *Acinetobacter calcoaceticus*, *Sphingobacterium multivorum*, *Enterobacter aerogenes*, *Bacillus cereus/anthracis*, *Yersinia pseudotuberculosis/pestis/enterocolitica* (no occurrence in NPK soil) and *Mycobacterium fortuitum* (no occurrence in tubers of MF treatment). Additionally, the majority of the potential pathogens identified in tubers from fertilized soils also occurred in tubers from CF, including *Staphylococcus lugdunensis/aureus*, *Stenotrophomonas maltophilia*, *Brucella suis/canis/abortus/melitensis* and *Klebsiella oxytoca/Enterobacter aerogenes*. Only a few ASVs occurred in fertilized tubers and not in the control ones, indicating their possible transmission from soil. Those included *Burkholderia pseudomallei*, *Nocardia brasiliensis*, *Aeromonas veronii*/*hydrophila*/*caviae* and *Serratia marcescens*. Importantly, none of the pathogens found in any fertilized soils were uniquely found in the tubers originated in that soil, indicating that no transfer of pathogens from organically fertilized soil into tubers took place.

## 4. Discussion

This study was focused on the influence of 21 years of chemical and organic fertilization on soil and endophytic microbial communities. The experiment was established in two geochemically and geographically distinct sites, hence, with different environmental conditions and edaphic characteristics (Table 1). The diversity of soil prokaryotes and fungi did not significantly differ between any of the fertilized soils and the CF. Only the soil fungal diversity was significantly higher with the MF treatment compared to SF, probably due to the introduction of a wide range of biopolymers promoting fungal diversity, as was reported previously [46,47]. Higher fungal diversity positively affects the ability of soil to suppress diseases [46], indicating that MF treatment is more likely to suppress soil-borne pathogens than other treated soils, as suggested by the lower relative abundance of the common soil-borne pathogen *Fusarium* [3] in tubers from MF-treated soil compared to control (Figure S3). In fact, the disease suppression does not generally require the complete eradication of the pathogen, while the establishment of a healthy and diverse microbiome can reduce the chances of infection or stimulate plant defenses [48], which might have been the case with MF treatment.

Soil microbial diversity has not always been reported to be significantly associated with fertilizer application, but to the best of our knowledge, changes in soil characteristics caused by fertilization have been reflected in the structure of the soil microbial community [13,49]. In this study, the soil community structure of both prokaryotic and fungal communities were significantly associated with fertilization treatments (Figure 3, Table 2), which is in agreement with previous findings; these findings reported either an indirect influence of fertilization on the soil community structure through the alteration of physicochemical soil properties [10,11], or direct influence through the introduction of new species into the soil by the application of organic fertilizers [27].

The relative abundance of 20 bacterial genera and seven fungal genera was identified in this study to be increased by at least one fertilization treatment (MF, SF, SF3x and/or NPK) compared to CF. Interestingly, all 20 of the bacteria were enriched in SF-treated soil (Table 2). Among the differently abundant genera, the genera *Turicibacter*, *Rhodanobacter*, *Flavobacterium*, *Clostridium*, *Pseudoxanthomonas* and *Coprothermobacter* have been previously isolated from sewage sludge or sewage sludge-treated soils [28,50,51,52], suggesting their possible direct transmission from the fertilizer to the soil. Several other differently abundant genera, such as *Hydrogenophaga* and *Flavobacterium*, were previously associated with the bioremediation of polluted soils [53,54,55], which corresponds to the findings that persistent organic pollutants, micropollutants and heavy metals are commonly found in sewage sludge [56,57], including the one used in this study [58]. Most of all, beneficial plant-associated genera were found to be significantly enriched in the SF and SF3x treatment, including *Stenotrophomonas* and *Sphingomonas*. *Stenotrophomonas* promotes the growth of plants and helps to control the abundance of fungal phytopathogenic fungi in soil through the production of chitinases [59,60]. *Sphingomonas* was found to be a keystone genus in healthy soils, and was also significantly associated with plant pathogen suppression [48]. Several plant-beneficial fungi were also identified to be significantly enriched by some of the fertilization treatments. *Funneliformis* and *Diversispora* (of the phylum Glomeromycota), which were enriched in MF, NPK and SF-treated soils, are arbuscular mycorrhizal fungi (AMF) [61], which enhance the solubility and availability of a wide range of nutrients, improve the soil structure, increase water uptake for plants and give them protection [62,63]. Their abundance in soil was previously associated with the fertilization regime and a higher uptake of macro- and micronutrients in plants [47]. SF-treated soil was also associated with a higher relative abundance of *Mortierella*, which is a phosphate-solubilizing fungal saprotroph whose presence positively influences the colonization of soil by AMF, and which was associated with higher plant weight [64]. An increased relative abundance of this genus associated with fertilization has already been reported [13]. *Basidiobolus*, a fungal genus enriched in MF- and SF-treated soils, is well-known for the production of chitinase, and therefore, *Basidiobolus* has been suggested to be used as a biocontrol for phytopathogenic fungi [65,66]. Only one genus among the differently abundant genera, *Conocybe*, which was enriched in NPK soils, was found to be potentially pathogenic [67].

In comparison with fertilized soils, the relative abundance of several prokaryotic and fungal genera was higher in CF (Figures S1 and S2). Prokaryotes, such as *Brevundimonas* and *Limnohabitans*, were mostly reported to be ubiquitous [68,69], whereas *Massilia* and *Phenylobacterium* were associated with biodegradation activity [30]. Several of these fungal genera are among agronomically promising taxa. *Coprinellus* is a fungal genus that was found to suppress rot diseases of vegetables [70]. *Idriella* is a biocontrol agent of take-all diseases of wheat and barley, and its presence in soil reduces the severity of the crop damage [71,72]. In our experiment, the potato was rotated with spring barley and winter wheat, thus, the lower relative abundance of *Idriella* in fertilized soils may potentially increase the risk of crop damage unless the lower relative abundance of *Idriella* is associated with the promotion of other plant-beneficial genera. On the other hand, CF treatment was found to contain a significantly higher relative abundance of *Ceratocystis*, a genus that includes many plant pathogens, such as *Ceratocystis fimbriata*, which causes rot of sweet potatoes [73], when compared to SF3x treatment.

Since our results showed that fertilization significantly influences the structure of soil microbial communities with an effect on the relative abundance of several beneficial microbes, we hypothesized that the application of fertilizer would also have an impact on the microbial community of endophytes in tubers. Understanding how fertilization influences endophytes is crucial for sustainable agriculture, since the manipulation of the plant microbiome could affect the plant benefits associated with the activity of endophytes [15,18].

As our results showed, site characteristics, fertilization and their interaction significantly shaped both soil and endophytic community structures. Whereas site characteristics had a higher influence on soil microbial communities than fertilization regimes, the endophytes were more influenced by fertilization and the interaction of site characteristics and fertilization (Table 2, R^2^ values), yet the specific influence of fertilization varied between the sites (Figure 3). In Humpolec, the prokaryotic and fungal endophytic communities significantly differed in their responses to the fertilization treatments, but the communities were not significantly associated with the fertilization treatment in Suchdol (Figure 3). This difference can be ascribed to the soil characteristics in each locality. Whereas the soil in Humpolec is a cambisol, which is one of the most widespread soil types [74], Suchdol has chernozem, which is one of the most fertile soils [74], and, compared to cambisol, has a higher content of oxidizable carbon and higher pH (Table 1) and in general higher microbial biomass and diversity [75]. Since plants recruit the endophytes to obtain growth and health benefits [76], we assume that the establishment of endophytic communities in this highly fertile and microbically diverse soil, is much less prone to changes brought about by fertilization.

The significant relationship between endophytic community structure and the fertilization regime was more pronounced for prokaryotes (*P_adj_* ≤ 0.001) than for fungi (*P_adj_* ≤ 0.1). Prokaryotic community structure significantly differed in MF and SF3x treatments compared to the CF in both sites. Prokaryotic endophytes were previously found to be significantly altered by the fertilization regime [24,69]. Specifically, methanogenic archaea increased with a higher application of N [21], or organic fertilizers enhance the number of diazotrophic bacteria [25]. The possible reason why MF and SF3x treatments were associated with major shifts in prokaryotic endophytic communities might not be only the new sources of nutrients, but primarily the transfer of these fertilizer-borne taxa into the soil [27,28] and their penetration into the endosphere. These inoculated microbes could be already present in the soil, or new taxa could be introduced. Although not all organic fertilizer-borne taxa can be recruited as plant endophytes, they can still influence the indigenous endophytic community structure [77].

Differential analysis allowed us to identify endophytes, whose relative abundance differed with fertilization. Four genera were found to be relatively enriched in tubers grown in fertilized soils (Table 3). Compared to CF, *Xylophilus* was enriched with MF and SF3x treatments, *Duganella* with NPK and SF3x and *Acinetobacter* with NPK treatment. Whereas *Acinetobacter* and *Duganella* are plant-growth promoting bacteria [78,79], *Xylophilus* is a phytopathogen. However, this bacterial genus only causes disease in grapevines [80] and its role in *Solanum tuberosum* L. remains unclear.

The increased use of organic fertilizers in agriculture has raised concerns about their safety [81]. Organic fertilizers can also introduce a range of human and animal pathogens into soil [82]. For instance, animal manures were found to increase the abundance of *Escherichia coli* O157:H7 [83,84] or *Listeria monocytogenes* in cultivated vegetables [85]. Such findings would imply a potential threat to human health. In this study, 22 ASVs were assigned to one or more potential human pathogens. However, the majority of the potential human pathogens identified were found in soil and tubers under all treatments, or they were also present in CF-soil or CF-tubers. Their occurrence in CF implies that they are part of indigenous microbial communities regardless of fertilization. Appropriate treatment of organic wastes, composting of animal manure and application timing can reduce the potential risk of microbial contamination [84]. Hence, our results do not indicate that either stabilized sewage sludge (at 55 °C) or manure produced by proper composting, both applied in the autumn, would pose a significant threat to human health.

In summary, our study contributes to the understanding of how microbial diversity and community structure in the soil and endosphere of *Solanum tuberosum* L. respond to 21 years of regular fertilization. Our results showed that while the soil prokaryotic and fungal communities are influenced by fertilization treatments, the effect of fertilization on endophytic communities is site-specific. Furthermore, the application of either chemical or organic fertilizers influenced the relative abundance of several plant-beneficial microbes in soil. In our study, we did not record the transfer of pathogenic microorganisms if properly stabilized manure and sewage sludge are used. What remains to be investigated is the succession of endophytic communities in fertilizer-treated soils over time, and there is a need to broaden the results to other types of soil and host-plant models.

## Figures and Tables

**Table 1:** Description of experimental sites.

	Site 1: Humpolec	Site 2: Prague-Suchdol
GPS	49°33′16″ N, 15°21′2″ E	50°7′40″ N, 14°22′33″ E
Elevation [m]	525	286
CEC ^1^ [mmol_(+)_/kg]	90	262
C_ox_ [%]	1.24	1.76
pH	5.27 ± 0.5	7.8 ± 0.4
Bulk density [g/cm^3^]	1.40	1.43
Clay [%]	5.84	2.18
Silt [%]	43.55	71.8
Sand [%]	50.61	26.03
Soil type (WRB 2006)	Cambisol	Chernozem
NRCS ^2^ USDA	silty loam	silty loam

^1^ CEC, Cation exchange capacity; ^2^ NRCS, Natural resources conservation service.

**Table 2:** Influence of fertilization regime, site characteristics and their interaction on the structure of prokaryotic and fungal communities originating from soil or potatoes (PERMANOVA). Significant (*P_adj_* ≤ 0.05) *P* values after the false discovery rate (FDR) correction are labeled with an asterisk (*).

	Bulk Soil				Tubers			
	Prokaryotes		Fungi		Prokaryotes		Fungi	
	R^2^	*P_adj_*	R^2^	*P_adj_*	R^2^	*P_adj_*	R^2^	*P_adj_*
**Site characteristics**	57%	0.001 *	39%	0.001 *	10%	0.001 *	7%	0.001 *
**Fertilization**	11%	0.002 *	14%	0.003 *	14%	0.001 *	11%	0.087
**Fertilization X Site char.**	8%	0.004 *	11%	0.007 *	18%	0.001 *	19%	0.001 *

**Table 3:** Results of differential abundance analysis showing bacterial or fungal genera significantly enriched in one or more fertilized variants compared to control treatment (CF). The results report on: (**A**) bacterial genera enriched in fertilized soils, (**B**) fungal genera enriched in fertilized soils, (**C**) bacterial genera enriched in tubers grown in fertilized soils. The fertilization variants tested were: manure (MF; 330 kg N/ha), NPK (NPK; 330-90-330 kg/ha), sewage sludge (SF; 330 kg N/ha), sewage sludge (SF3x; 990 kg N/ha).

(A) Significantly Enriched Bacteria in Fertilized Soil		
Fertilization	Total	Genera
MF SF SF3x	2	*Arenimonas*, *Herminiimonas*
NPK SF SF3x	2	*Nitrosospira*, *Rhodanobacter*
SF SF3x	12	*Turicibacter*, *Ferruginibacter*, *Hydrogenophaga*, *Flavobacterium*, *Stenotrophomonas*, *Actinomadura*, *Devosia*, *Sphingomonas*, *Chitinophaga*, *Clostridium sensu stricto* 1, *Chryseolinea*, *Aminobacter*
NPK SF	1	*Granulicella*
SF	3	*Candidatus*_*Caldatribacterium*, *Pseudoxanthomonas*, *Coprothermobacter*
**(B) Significantly Enriched Fungi in Fertilized Soil**		
**Fertilization**	**Total**	**Genera**
MF NPK SF SF3x	2	*Apodus*, *Operculomyces*
MF NPK SF	2	*Funneliformis*, *Diversispora*
MF SF	1	*Basidiobolus*
SF	1	*Mortierella*
NPK	1	*Conocybe*
**(C) Significantly Enriched Bacteria in Tubers Planted in Fertilized Soil**		
**Fertilization**	**Total**	**Genera**
MF SF3x	1	*Xylophilus*
NPK SF3x	1	*Duganella*
NPK	1	*Acinetobacter*

## Supplementary Materials

The following are available online at https://www.mdpi.com/2076-2607/8/9/1377/s1, Figure S1: Pairwise comparison (DeSeq analysis) of the abundance of soil prokaryotic genera with significant (*P_adj_* < 0.01) difference between control soil (CF) and one of the fertilization treatment: manure (MF; 330 kg N/ha), NPK (NPK; 330-90-330 kg/ha), sewage sludge (SF; 330 kg N/ha), sewage sludge (SF3x; 990 kg N/ha). Negative “log2 Fold Change” values (*x*-axis) indicate a higher abundance of genera in fertilized soils and positive values indicate higher abundances in CF. Figure S2: Pairwise comparison (DeSeq analysis) of the abundance of soil fungal genera with significant (*P_adj_* < 0.01) difference between control soil (CF) and one of the fertilization treatment: manure (MF; 330 kg N/ha), NPK (NPK; 330-90-330 kg/ha), sewage sludge (SF; 330 kg N/ha), sewage sludge (SF3x; 990 kg N/ha). Negative “log2 Fold Change” values (*x*-axis) indicate a higher abundance of genera in fertilized soils and positive values indicate higher abundances in CF. Figure S3: Pairwise comparison (DeSeq analysis) of the abundance of prokaryotic (A) and fungal (B) genera isolated from potato tubers with a significant (*P_adj_* < 0.01) difference between control treatment (CF) and one of the fertilization variant: manure (MF; 330 kg N/ha), NPK (NPK; 330-90-330 kg/ha), sewage sludge (SF; 330 kg N/ha), sewage sludge (SF3x; 990 kg N/ha). Negative “log2 Fold Change” values (*x*-axis) indicate a higher abundance of genera in fertilized soils and positive values indicate higher abundances in CF.
